# Gender differences in self-harm and drinking behaviors among high school students in Beijing, China

**DOI:** 10.1186/s12889-020-09979-6

**Published:** 2020-12-09

**Authors:** Chai-Quan Li, Jing-Shu Zhang, Shang Ma, Ruo-Ran Lv, Jia-Li Duan, Dong-Mei Luo, Xiao-Jin Yan, Ning Ma, Yi Song

**Affiliations:** 1 School of Public Health, Peking University, Beijing, China; 2 Institute of Child and Adolescent Health, School of Public Health, Peking University, Beijing, China; 3 The School of Health Humanities, Peking University, Beijing, China; 4 Beijing Center for Disease Prevention and Control, Beijing, China

**Keywords:** Self-harm, Drinking, High school students, Association

## Abstract

**Background:**

Self-harm and drinking are both serious problems in adolescents and many studies presented evidence of their association. However, gender differences in this association are seldom deeply discussed. Our study aimed to evaluate the prevalence of self-harm and explore its association with drinking behaviors by gender and investigate the extent to which the gender differences exist in the association between self-harm and drinking.

**Methods:**

A total of 32,362 students in grades 7 to 12 in Beijing, China were anonymously surveyed and included in our study using two-stage, stratified probability proportion sampling. Self-harm, drinking behaviors and other basic information were obtained from an anonymous questionnaire. Demographic variables, self-harm and drinking behaviors were analyzed using the Chi-square test and the Gamma test between genders and the gender differences in this association were analyzed by Log-binomial regression.

**Results:**

The total prevalence of self-harm was 13.7% with no significant gender difference (*χ*^2^ =0.352, *P* = 0.553). The prevalence of self-harm in girls decreased with age (*G* = -0.163, *P* < 0.001). Self-harm was associated with drinking behaviors in both boys and girls. The Log-binomial regression demonstrated that girls in the 16–19 years old group were at lower risk of self-harm than girls in the 12–15 years old group while this association was weaker in boys (1.493 vs 1.128). The higher OR for self-harm was found among girls with early drinking experiences compared with boys (2.565 vs 1.863). Girls who had previously drunk (i.e. drunk at least once) (2.211 vs 1.636), were currently drinking (3.400 vs 2.122) and performed binge drinking (6.357 vs 3.924) were at greater risk of self-harm than boys.

**Conclusion:**

Among high school students, self-harm has a significant positive association with drinking and girls with drinking behaviors are at higher risk of suffering self-harm. Identifying adolescents’ drinking behaviors is of vital importance to self-harm prevention and special attention should be focused on younger girls.

## Background

Self-harm is defined as a person’s harming of his or her own body on purpose such as self-injury and self-poisoning, irrespective of the motive or the extent of suicidal intent [1–5]. Self-harm causes a great health expenditure and loss for health care resource [6]. It is associated with numerous somatic diseases [7], in particular, with mental or psychiatric diseases such as depression and anxiety [5, 8]. Within our life span, self-harm is the most prevalent during puberty [9]. Globally, the prevalence of self-harm ranged from 3.1 to 15.5% among adolescents aged between 12 to 21 years [3, 4, 10–15], while in China, the prevalence reached 27.6% and some subtypes of self-harm were as high as 32.0% [5, 16]. This higher self-harm prevalence might be mainly interpreted by academic-related stress due to the competitiveness in the education system [17]. Some studies about suicide also supported that it was the heavy academic burden that resulted in the high suicide prevalence in Chinese [18] or in Asian American [19] groups.

An ‘iceberg model’ is often raised to describe the situation of suicide, which also reflects self-harm as it is recognized as a ‘*hidden behavior*’ whereby less than 13% of the self-harm episodes led to hospital presentation [2, 7, 15]. 55.8% of the subjects who had committed self-harm in a cohort study did not refer to mental health services [8]. Thus, clinical based investigation might underestimate the prevalence of self-harm and conducting a self-reported survey might help us find those who have committed self-harm but without hospitalization. Moreover, hidden behavior means that we need more readily observed markers or indicators to help identify self-harm.

Drinking is also a serious public health concern in adolescents and the rate of current drinking (alcohol use in the past 30 days) among Chinese adolescents ranged from 7.3 to 25.2% and the rate of alcohol ever use ranged between 50.9 and 54.1% [20–23]. Among Arab Muslims the prevalence of alcohol use in the past year was 9.6% [24] compared with 39.8% [22] among Chinese adolescents. However, alcohol use was still common among adolescents in many developing countries such as Brazil [25], Mexico [26] and Chile [27]. Also, according to the Global Status Report on Alcohol and Health published by World Health Organization, the prevalence of women’s current drinking in Western Pacific regions was not decreasing with other regions of the world. In Western Pacific regions, the prevalence of women’s current drinking has been still over 40% by 2016 [28]. Moreover, drinking is not only a prevalent behavior in adolescents but also an observable behavior. Numerous studies have reported the positive association between self-harm and drinking (or alcohol abuse) [3, 7, 8, 10, 12, 15, 29–36] . Ness et al. conducted an epidemiological and longitudinal study and found that alcohol was involved in 58.4% of self-harm episodes [36]. Heerde et al. found that recent alcohol users were 2.70 times at risk to commit self-harm [33]. However, most studies focused on the relationship between current drinking condition and self-harm. Whether gender disparity exists in the association between self-harm and drinking needs further study. Limited studies showing gender differences in the relationship between self-harm and drinking behaviors demonstrated that the association of current drinking condition with self-harm was more significant among girls [34], although it was only a rather small part of the results and was not fully discussed. Other studies identified that the early onset of alcohol use could be a risk factor for self-harm but did not discuss the gender difference in the relationship between self-harm and early drinking experience [30, 33]. Another study even found that alcohol-related self-harm was more common in men rather than women though its subjects were mainly adults [36]. As such, the gender differences in this association require deeper research. We hypothesize that among adolescents, the association of self-harm and drinking behaviors is stronger in girls than boys. The same factors can bring more risk to girls.

China is now undergoing an epidemiological change in which the main health burden of adolescents is switching from communicable diseases and undernutrition to non-communicable diseases and especially, mental health disorders [37]. During the past 12-months the prevalence of self-harm was higher in low- and middle-income countries than developed countries [17]. Also, total alcohol consumption per capita increased in the Western Pacific region and South-East Asia region, while women’s drinking in both regions remained stable. Our study may provide evidence for those low- and middle-income countries which were undergoing the same process as China [28]. Therefore, this study aimed to evaluate the prevalence of self-harm and explore its association with drinking behaviors by gender and investigate the extent to which the gender differences existed in the association between self-harm and drinking.

## Methods

### Design and sampling

**Fig. 1:**
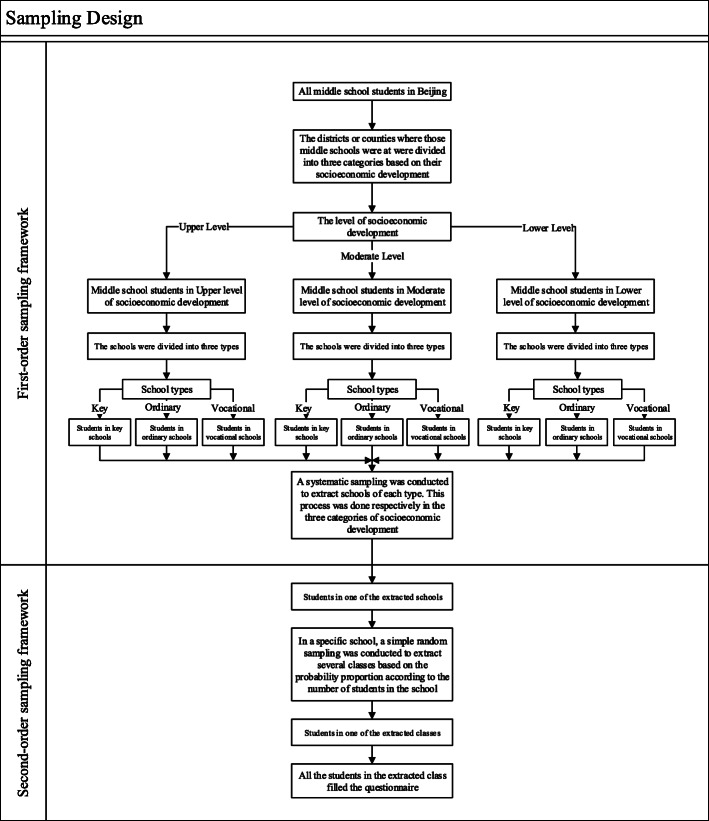
The sampling process of Beijing Youth Health Risk Behaviors Survey, 2014

A two-stage, stratified probability proportion sampling was conducted to obtain a representative sample for high school students in Beijing, China. The first stage of the sampling was to extract schools which were classified based on socioeconomic development levels of the districts or counties in which those high schools were located and based on whether the school was a ‘*Key school’* (referring to a school with good records of past educational accomplishment and had priority in the assignment of teachers, equipment and funds, and with the privilege of recruiting the best students [18]). The levels of socioeconomic development were classified as *upper, moderate* and *lower* according to the local economic development. At the start, all high schools in Beijing formed as the first-order sampling framework. A probability proportion sampling was conducted to extract schools stratified by school type, which was done for each of the three categories of socioeconomic development. The second stage of the sampling was to extract students in the selected schools. Random sampling by grade was conducted by using class as the sampling unit with all students in the sampled class participated in the survey (see Fig. 1). The investigation was conducted from April to May, 2014. Each participant in this survey was required to complete a self-reported anonymous questionnaire in absence of his or her teacher.

### Measures

The questionnaire was derived from the 2003 Youth Risk Behavior Surveillance survey in the United States [38], which was a widely accepted questionnaire with robust reliability and validity [39–41].

#### Self-harm

The frequency of self-harm behaviors was asked in the questionnaire as *“In the past 12 months, have you ever deliberately committed self-harm (such as burning oneself, cutting oneself and hitting the wall with one’s head etc..)”*. Many scales or studies for estimating self-harm adopted similar questions to determine whether the participant had the experience of self-harm [8, 10, 42], and its reliability and validity were already examined to be qualified [43]. The choices for this question include *never*, *once*, *twice or three times* and *four times or more*.

#### Drinking behaviors

Two variables of drinking behaviors were adopted to look into the association between different aspects of drinking behaviors and self-harm. The age of one’s first drinking was asked by “*How old were you when you had your first drink of alcohol other than a few sips?*” Having one’s first drink before 13 years old was considered as early drinking experience [40]. The condition of drinking behaviors was defined as: lifetime (at least one previous drink), current (at least one alcoholic drinks in the past 30 days) and binge drinking (at least five alcoholic drinks per occasion in 1 day during the past 30 days) [38, 44]. These definitions were used in previous studies [23, 33]. A recoding process referring to Heerde et al.’s research [33] was utilized to simplify four variables that described drinking conditions of participants into one variable with four categories, defined as *no alcohol use*, *non-recent alcohol use* (lifetime use but no use in the past month), *recent alcohol use* (use in the past month but no binge use), and *binge drinking* (binge alcohol use in the past month). The initial questions for the four categories were as follows, “*Have you ever had your first drink other than a few sips?*”; “*In the past 30 days, on how many days did you have at least one drink containing alcohol?*” and “*During the past 30 days, on how many days did you have ≥ 5 drinks of alcohol in a row?*”

#### Controlling variables

Four basic demographic variables were controlled in the analysis for the association between self-harm and drinking behaviors, which included gender, age, urban or suburban area and school type (*Key schools* or *Non-Key schools)*.

### Statistical analysis

**Fig. 2:**
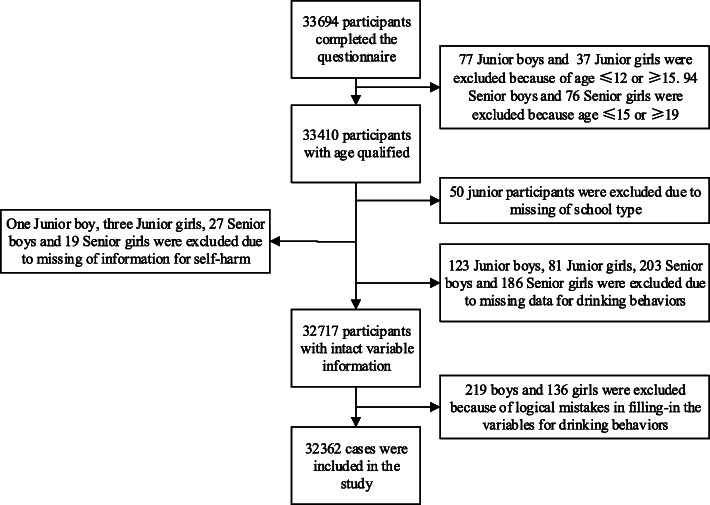
The participants included in our study

A total of 33,694 high school students completed the questionnaire (including 16,819 girls). One thousand three hundred thirty-two participants were excluded due to missing information, logic error or unqualified age. The response rate of self-harm was 99.9% (33,644 in 33,694) and of drinking behaviors was 98.2% (33,081 in 33,694). No differences were found between the excluded participants and the remaining sample in gender and age. The final sample size was 32,362. The number of participants included in our study are shown in Fig. 2.

A descriptive analysis was conducted to present the general information of the sample and the association between self-harm and drinking behaviors. The Chi-square test was used to compare the difference between non-ordered groups such as whether the subjects committed self-harm in different gender groups. The Gamma test was used to compare self-harm frequency, which was an ordered variable, between different age groups, different settings and different school types. A Log-binomial regression was conducted to test the differences of the association between self-harm and drinking behaviors by gender. To be prudent, three models for different risk factors of self-harm were established due to the correlations between each other. All analyses were executed by gender. The gender disparity in the association between self-harm and drinking behaviors were evaluated by adding one interaction term of drinking behaviors and gender in each model. Significance level was accepted at *P* < 0.05, two-tailed. All data were analyzed using SPSS20.0 for Windows (SPSS Inc., Chicago, IL, USA) or Stata 15 SE (Stata Corp LLC).

## Results

The final sample size was 32,362, including 16,103 boys (49.8%), 16,241 urban students (50.2%), 12,065 Key school students (37.3%) and 15,672 12 ~ 15 years old (48.4%).

### The prevalence of self-harm by gender

**Fig. 3:**
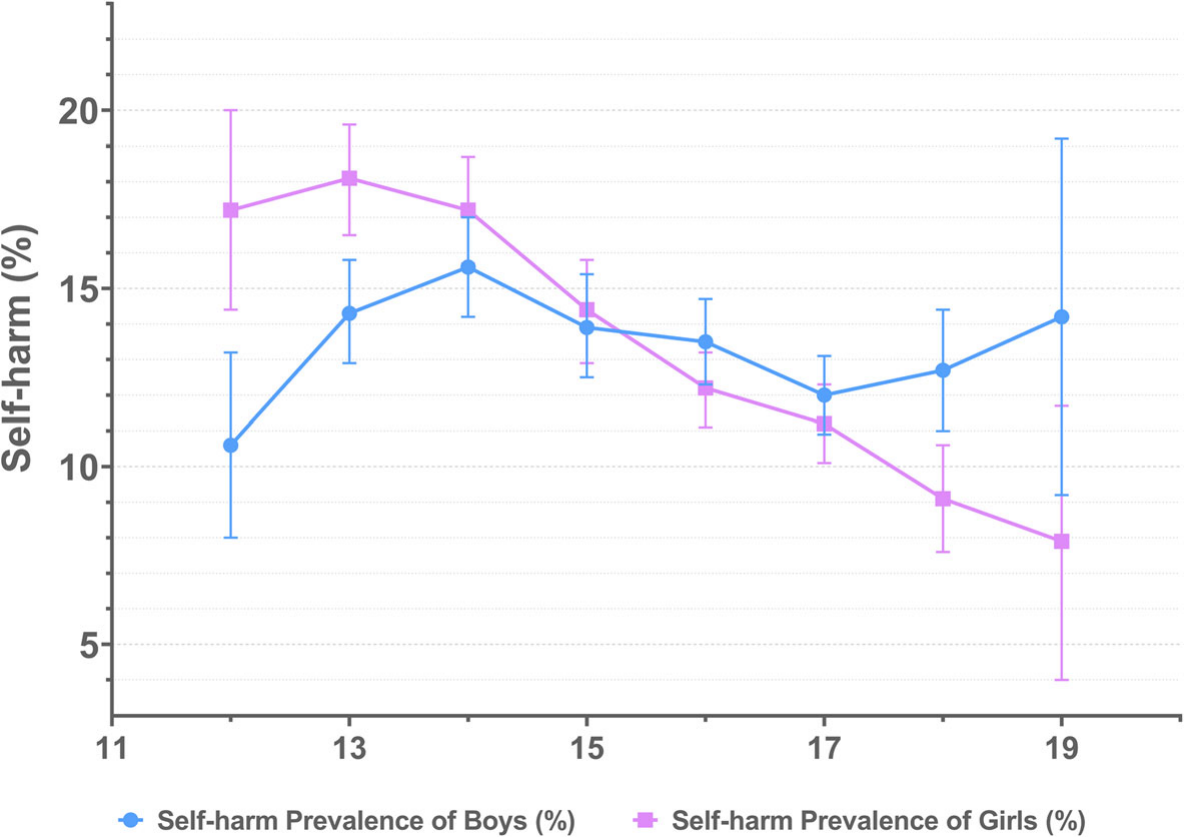
The prevalence of self-harm by age

The total prevalence of self-harm was 13.7%. Among all surveyed students, 7.1% reported that they had self-harmed more than once. No significant difference was found in the prevalence of self-harm between genders (*χ*^2^ =0.352, *P* = 0.553). Totally, the prevalence of self-harm decreased with age in girls (*G* = -0.163, *P* < 0.001) but the prevalence of self-harm in boys seemed to be fluctuating with age (See Fig. 3), and despite this the association in boys was still statistically significant (*G* = -0.038, *P* = 0.010). The gender differences existed in both younger and older adolescence, but with different directions, within the adolescents younger than 16, the prevalence of self-harm was higher in girls than boys (*χ*^2^ =18.388, *P* < 0.001), while the boys showed higher prevalence than girls when they were in older adolescence (*χ*^2^ =6.870, *P* = 0.009). Girls who were in Key schools were less vulnerable to self-harm compared with those in non-Key schools (*G =* 0.075, *P =* 0.001) but the prevalence showed no difference between school types among boys (*G =* 0.023, *P =* 0.306). The prevalence of self-harm in different demographic groups was shown in Table 1.

**Table 1:** Self-harm prevalence in different demographic groups

	Total	Frequency of self-harm (in past 12 months)				*G*	*P*
		Never	Once	Twice or three times	Four times or more		
Boys							
Age							
12 ~ 13	2942	2540 (86.3)	198 (6.7)	110 (3.7)	94 (3.2)	−0.041	0.007
14	2698	2277 (84.4)	202 (7.5)	127 (4.7)	92 (3.4)		
15	2212	1904 (86.1)	145 (6.6)	90 (4.1)	73 (3.3)		
16	3348	2896 (86.5)	200 (6.0)	128 (3.8)	124 (3.7)		
17	3246	2857 (88.0)	181 (5.6)	118 (3.6)	90 (2.8)		
18 ~ 19	1656	1443 (87.1)	84 (5.1)	70 (4.2)	59 (3.6)		
Setting							
Urban	8100	7005 (86.5)	495 (6.1)	323 (4.0)	277 (3.4)	0.002	0.920
Suburban	8003	6913 (86.4)	515 (6.4)	320 (4.0)	255 (3.2)		
School type							
Key School	5761	5001 (86.8)	352 (6.1)	216 (3.7)	192 (3.3)	0.023	0.306
Non-Key School	10,342	8917 (86.2)	658 (6.4)	427 (4.1)	340 (3.3)		
Total	16,103	13,918 (86.4)	1010 (6.3)	643 (4.0)	532 (3.3)		
Girls							
Age							
12 ~ 13	3106	2551 (82.1)	275 (8.9)	175 (5.6)	105 (3.4)	−0.167	< 0.001
14	2468	2044 (82.8)	192 (7.8)	156 (6.3)	76 (3.1)		
14	2246	1923 (85.6)	167 (7.4)	106 (4.7)	50 (2.2)		
15	3559	3126 (87.8)	231 (6.5)	135 (3.8)	67 (1.9)		
17	3202	2844 (88.8)	182 (5.7)	121 (3.8)	55 (1.7)		
18 ~ 19	1677	1527 (91.1)	72 (4.3)	51 (3.0)	27 (1.2)		
Setting							
Urban	8141	6996 (85.9)	557 (6.8)	386 (4.7)	202 (2.5)	−0.024	0.274
Suburban	8118	7020 (86.5)	562 (6.9)	358 (4.4)	178 (2.2)		
School type							
Key School	6304	5508 (87.4)	381 (6.0)	269 (4.3)	146 (2.3)	0.075	0.001
Non-Key School	9955	8508 (85.5)	738 (7.4)	475 (4.8)	234 (2.4)		
Total	16,259	14,016 (86.2)	1119 (6.9)	744 (4.6)	380 (2.3)		

### Univariate analysis of self-harm and drinking behaviors by gender

**Fig. 4:**
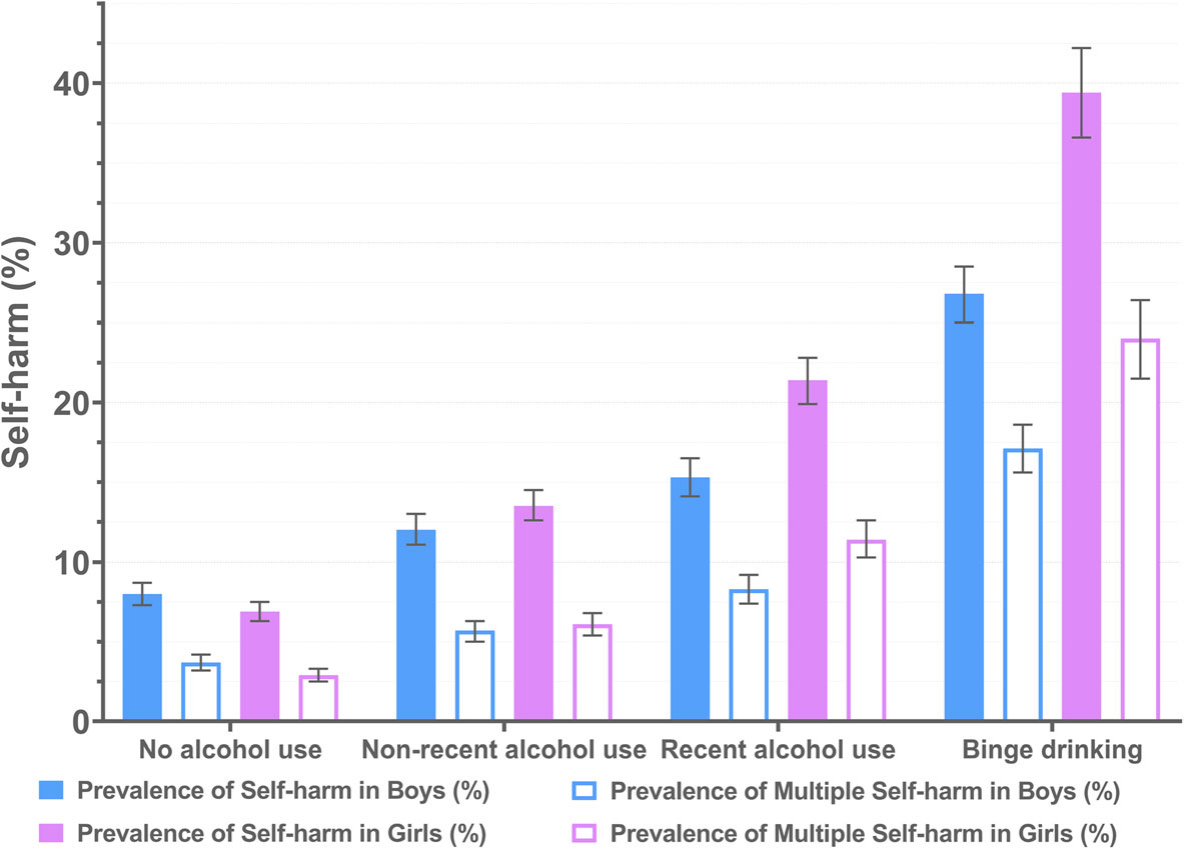
The prevalence of self-harm and multiple self-harm in different groups of drinking condition by gender

Boys were more likely to have early drinking experience than girls (41.8% in boys and 31.2% in girls, *χ*^2^ =392.253, *P* < 0.001) and drinking behaviors were more prevalent among boys (23.1% of the boys reported recent alcohol use while 13.4% of the girls, *χ*^2^ =299.677, *P* < 0.001, see Supplementary Table 1). Drinking behaviors were associated with self-harm in both boys and girls. The frequency of self-harm was higher in early drinkers (Boys: *G* = 0.338, *P* < 0.001; Girls: *G* = 0.507, *P* < 0.001). The more severe the extent of drinking condition was, the more frequency of self-harm was found in both boys (*G* = 0.345, *P* < 0.001) and girls (*G* = 0.475, *P* < 0.001). Among those girls who were current drinkers and binge drinkers, the prevalence of self-harm was as high as 21.4% and 39.4% respectively (see Fig. 4). 24.0% of the girls with binge drinking experience reported multiple self-harm in the past 12 months, indicating a much higher prevalence than those without binge drinking experience (*χ*^2^ =572.930, *P* < 0.001).

### Gender disparity in the association between self-harm and drinking behaviors

The Log-binomial regression demonstrated that girls in earlier adolescence (12–15 years) were at higher risk of self-harm than girls in older adolescence (16–19 years) but this association was not that strong in boys. Participants with early drinking experiences had a higher risk for self-harm compared to those who did not (AOR = 1.854). Higher AOR for self-harm was found among girls with early drinking experiences (2.565 vs 1.863). Both genders with any extent of drinking condition presented higher risk for self-harm. Girls who had previously drunk (i.e. drunk at least once) (2.211 vs 1.636), were currently drinking (3.400 vs 2.122) and binge drinking (6.357 vs 3.924) were of higher risk for self-harm than boys. All the AORs for self-harm mentioned above were shown in Table 2.

**Table 2:** Association analyses between self-harm and drinking behaviors (include age)

Risk Factors	Total	Girls	Boys	Interaction term^d^ *p*
	AORs	AORs	AORs	
Age^a^				
12 ~ 15	1.311 (1.240, 1.385)	**1.507 (1.394, 1.629)**	**1.134 (1.041, 1.227)**	**< 0.001**
Drink condition^b^				
Non-recent alcohol use	1.680 (1.492, 1.892)	**2.211 (1.979, 2.471)**	**1.636 (1.451, 1.844)**	**0.002**
Recent alcohol use	2.187 (1.942, 2.464)	**3.400 (3.054, 3.786)**	**2.122 (1.882, 2.393)**	**< 0.001**
Binge drinking	4.106 (3.670, 4.594)	**6.357 (5.707, 7.080)**	**3.924 (3.498, 4.401)**	**< 0.001**
Age of First Drink^c^				
< 13	1.854 (1.714, 2.006)	**2.565 (2.376, 2.770)**	**1.863 (1.721, 2.016)**	**< 0.001**

^a^ Controlling settings and school type, compared with the 16 ~ 19 age group

^b^ Controlling setting, school type and age, compared with *no alcohol use*. Drinking condition was a variable defined in the *Methods* section to describe the alcohol use of participants

^c^ Controlling setting, school type and age, compare with ≥13

^d^ Interaction between gender and the risk factors listed

## Discussion

We found obvious gender differences in the prevalence of self-harm in our study, in which during the earlier age, the prevalence of self-harm was higher in girls than boys while boys exceed girls in the older age. However, in whole age sample, the gender differences tended to disappear. The trends in self-harm prevalence of boys and girls were similar to Boeninger et al.’s study of suicide in Americans aged 11 ~ 19. In their study, the prevalence of suicide ideation and attempts was higher in girls of earlier age and boys’ prevalence surpassed girls’ in late teenage. Puberty occurred earlier in girls mostly and this period could be a temporary risk factor for girls since some psychological disorders [45], such as depression [46] and anxiety [47] increased rapidly in girls of this period.

A developmental gap between puberty and brain development was assumed to exist as the prefrontal cortex, the part of one’s brain regulating cognitive control [48], was undergoing a thinning and structural transformation process during puberty [49]. This process was related to enhancing the efficiency of the communication between neurons, the stability and precision of the synapses of the prefrontal cortex and was more significant in girls than boys [49]. Because prefrontal cortex was responsible for generating and maintaining the ability to adopt cognitive tactics to reframe negative emotional stimuli [49] and controlling impulsive behaviors [50], it could be speculated that the obvious decreasing rate of self-harm in girls might be based on this special process. It is worth noting that heavy drinking could weaken prefrontal networks, disrupt cognitive performance and exacerbate impulsivity [50], which may partly explain that why drinking girls were more likely to commit self-harm since the maturation of prefrontal cortex may play an important role in protecting girls from losing control of their behaviors. However, boy drinkers tend to manifest aggressive behaviors such as fighting [51].

Our results demonstrated that the association between self-harm and drinking behaviors differed between genders. Drinking behaviors in girls seemed to have stronger association with self-harm than boys. Some studies on the metabolism of alcohol in adults manifested that the ability to eliminate intracorporal ethanol was limited in women compared with men. The alcohol dehydrogenase (ADH), which was the enzyme catalyzing the phase I ethanol metabolism reaction, was less active in women than men [52]. Also, the low affinity of gastric *χ*-ADH of women resulted in the enhanced vulnerability of women to develop alcohol-related disease [53]. Some genotypes of aldehyde dehydrogenase (ALDH) were earlier inactivated among women, which might lead to more serious intolerance of alcohol in women than men [52]. Ness et al. conducted a converse research which was aimed to investigate the prevalence of alcohol abuse in those who had committed self-harm. Among them, more men were involved in alcohol use but alcohol abuse and self-injury were associated more significantly in women. Alcohol abuse in women would increase the risk of death by subsequence suicide in women [36]. Moreover, manifestation in men and women could be different between acute alcohol users and the long-term users. Berman et al. adopted the Self-Aggression Paradigm, a laboratory analog of non-suicidal deliberate self-harm, to prospect the acute effect of alcohol on the occurrence of self-harm. In this research, men more readily exerted self-harm analogical behaviors than women and the extent of self-harm was dose-dependent to the concentration of blood ethanol [29]. This needed further study to determine the different time phase property of the association between self-harm and drinking behaviors.

China is a country where drinking is regarded as a kind of traditional culture. People drink when it comes to essential events from traditional festivals to commercial negotiations. For most adolescents, drinking alcohol was sometimes encouraged by their parents for its function of social communication in Chinese culture. Among those who were high school students, the prevalence of alcohol use (at least once) was 51.1% [23]. Gender difference was still obvious pertaining to drinking behaviors. Boys were 1.78 times more likely to be current drinkers than girls and 1.86 times more likely to have alcohol related problems [20] but this did not mean that girls were safe. Drinking has been always inhibited or at least, not encouraged in girls from the cultural perspective. As a matter of fact, boys drink not only when they are suffering from negative emotions. In many other situations, they drink when they are happy, showing respect to others or just strutting their maturity. On the contrary, once girls frequently drink, it will be regarded as a risk for society’s traditional moral problems [54]. A possible explanation for the difference of the association between drinking and self-harm among boys or girls is that occasional heavy drinking is almost normative in men according to the social context factors while the girls who drank are likely to be those who have difficulty in adapting to their environments and more readily to have socio-psychological problems.

As our study presented, more serious drinking behaviors were associated with higher rate of self-harm. This phenomenon was significant in both boys and girls, with girls more significant than boys. Studies demonstrated that anxiety and depression were more prevalent in girls and both were associated with alcohol use disorder [55, 56]. In our study, the association between current drinking condition and the rate of self-harm may be dose-dependent. Though we did not find studies for adolescents to prove this relationship, Strine et al. conducted research for adults and one of their results concluded that the higher severity of depression would elicit more binge drinking among women but not men [57]. From another perspective, depression was more readily found in women who consumed larger quantities per drinking period instead of men [58]. Hawton et al. claimed in his review of self-harm in adolescents that anxiety and depression both were risk factors for self-harm [4]. Thus, a more serious drinking condition was associated with more prevalent of self-harm in both boys and girls while this possible dose-dependent effect was stronger in girls. This was also the reason why those who drank because of *feeling down*, or *habit* were the two groups of people committed the highest prevalence of self-harm (see Supplementary Table 2).

Early drinking experience was popular among the subjects in our research, which was consistent with another longitudinal study focusing on the effect of early sipping or tasting. Its evidence demonstrated that early sipping or tasting alcohol, even with parental permission, predicted increased frequency and quantity of alcohol consumption, and increased alcohol-related problems in late adolescence [59]. Non-Key school girls were vulnerable to self-harm but the association with drinking was not significantly different from that of boys. We built a regression model for school type (see Supplementary Table 3) and found that school type was not a risk factor in the model of the total sample. We believe its practical significance is limited.

Considering the significant association between self-harm and drinking behaviors, with the finding specific among younger girls, initiatives to prevent youth drinking may reduce self-harm among adolescents. The measures, such as legislating age restrictions on purchases of alcoholic beverages [28], I. D registration in the bar and combination of general and alcohol-specific parent-based interventions [60], may not only control drinking behaviors but also prevent self-harm. Moreover, psychological care should be provided to girls who are drinking, especially for younger girls. Liaison with psychiatry services may be helpful for protecting these girls against self-harm [35].

Our results may provide evidence for other countries with similar concerns. Some developing countries are facing comparable problems to China. For example, adolescent drinking was prevalent in Chile, which was found to be associated with many mental health problems [27]. Additionally, the prevalence of current drinking in women was still over 40% in the American region by 2016 despite experiencing a decrease [28]. Like China, women’s drinking did not significantly decrease in India [28] and although Indian research on self-harm was at an initial stage, one study had reported that the lifetime prevalence of non-suicidal self-injury was 17.2% [61]. Our study still has several limitations. Firstly, it was a cross-sectional study which could not determine the causal relationship between drinking and self-harm. Secondly, our study was based on a self-reported questionnaire which may be influenced by recall bias and reporting bias. However, the reliability and validity of our questionnaire has been proven and all processes were under strict control. Thirdly, some parental behavioral backgrounds, which may be associated with their offspring’s behavioral problems, were not included in our questionnaire. Finally, our study was based on the data collected from school samples, which may not represent those adolescents who dropped out of school. One global research on adolescents physical activity based on school-going adolescents admitted that collecting data from out-of-school adolescents was quite impossible and that this was a problem which needed to be urgently addressed [62]. Fortunately, China’s enrolment rate of primary school-age children in 2013 was 99.7%. The junior high school enrolment rate was 98.3% and 91.2% for senior high school [63]. Our sample not only contained those who were from vocational schools but also extracted from Beijing, the capital and the cultural center of China. These factors mean that our sample is still representative though no more than 10% of adolescents are not in our sampling frame.

## Conclusion

Our study presents that self-harm behavior was significantly positively associated with drinking behaviors among high school students. Self-harm might be identified by drinking behaviors, especially for girls. Although the prevalence of intemperate drinking behavior was lower in girls, the prevalence of self-harm was higher in those girls with active drinking condition. Moreover, it is important to focus on younger girls, who have the highest prevalence of self-harm. Interventions aimed at drinking behaviors might also be effective in preventing self-harm. Comprehensive action to prevent self-harm by identifying adolescents’ drinking behaviors will require engagement and coordinated responses across multiple stakeholders including, but not limited to, schools, families and community health workers.

## Supplementary Information

**Additional file 1: Supplementary Table 1.** Description of drinking behaviors and association with self-harm frequency by gender.

**Additional file 2: Supplementary Table 2.** Results of Log-binomial regression (Reasons for drinking).

**Additional file 3: Supplementary Table 3.** AORs for students in different school types by gender.

## Data Availability

The datasets generated and/or analyzed during the current study are not publicly available due to data management of Peking University, China, but are available from the corresponding author on reasonable request.

## Abbreviations

AOR: Adjusted odds ratio
ADH: Alcohol dehydrogenase
ALDH: Aldehyde dehydrogenase
