# Supplementary material for: Gender differences in self-harm and drinking behaviors among high school students in Beijing, China
Source: BMC Public Health. 2020 Dec 9;20:1892. doi: 10.1186/s12889-020-09979-6 (PMC7726872; doi:10.1186/s12889-020-09979-6)
Supplement: Supplementary file 1 — Additional file 1: Supplementary Table 1. Description of drinking behaviors and association with self-harm frequency by gender. [file 12889_2020_9979_MOESM1_ESM.docx]

Supplementary Table 1 Description of drinking behaviors and association with self-harm frequency by gender

|  |  |  | Drink Condition | | | | Total |
| --- | --- | --- | --- | --- | --- | --- | --- |
|  |  | Age of first drink <13 | No alcohol use | Non-recent alcohol use | Recent alcohol use | Binge drinking |  |
| Boys |  |  |  |  |  |  |  |
| Age group^a^ | 12~13 | 1266(43) | 1597(54.3) | 723(24.6) | 460(15.6) | 162(5.5) | 2942 |
|  | 14 | 1191(44.1) | 1190(44.1) | 748(27.7) | 498(18.5) | 262(9.7) | 2698 |
|  | 15 | 955(43.2) | 779(35.2) | 651(29.4) | 510(23.1) | 272(12.3) | 2212 |
|  | 16 | 1448(43.2) | 918(27.4) | 966(28.9) | 833(24.9) | 631(18.8) | 3348 |
|  | 17 | 1299(40) | 721(22.2) | 992(30.6) | 818(25.2) | 715(22) | 3246 |
|  | 18~19 | 569(34.4) | 349(21.1) | 529(31.9) | 384(23.2) | 394(23.8) | 1656 |
| Setting^a^ | Urban | 3544(43.8) | 2873(35.5) | 2319(28.6) | 1799(22.2) | 1109(13.7) | 8100 |
|  | Suburban | 3185(39.8) | 2681(33.5) | 2290(28.6) | 1705(21.3) | 1327(16.6) | 8003 |
| School type^a^ | Key school | 2374(41.2) | 2227(38.7) | 1704(29.6) | 1208(21) | 622(10.8) | 5761 |
|  | Non-Key school | 4355(42.1) | 3327(32.2) | 2905(28.1) | 2296(22.2) | 1814(17.5) | 10342 |
| Frequency of Self-harm^b, c^ | Never | 5477(81.4) | 5112(92) | 4054(88) | 2968(84.7) | 1784(73.2) | 13918 |
|  | Once | 545(8.1) | 234(4.2) | 294(6.4) | 246(7) | 236(9.7) | 1010 |
|  | Twice or three times | 368(5.5) | 119(2.1) | 159(3.4) | 166(4.7) | 199(8.2) | 643 |
|  | Four times or more | 339(5) | 89(1.6) | 102(2.2) | 124(3.5) | 217(8.9) | 532 |
| Total |  | 6729 | 5554 | 4609 | 3504 | 2436 | 16103 |
| Girls |  |  |  |  |  |  |  |
| Age group^a^ | 12~13 | 1174(37.8) | 1869(60.2) | 595(19.2) | 492(15.8) | 150(4.8) | 2942 |
|  | 14 | 894(36.2) | 1326(53.7) | 603(24.4) | 393(15.9) | 146(5.9) | 2698 |
|  | 15 | 734(32.7) | 998(44.4) | 651(29) | 436(19.4) | 161(7.2) | 2212 |
|  | 16 | 1064(29.9) | 1337(37.6) | 1203(33.8) | 718(20.2) | 301(8.5) | 3348 |
|  | 17 | 845(26.4) | 1225(38.3) | 1059(33.1) | 629(19.6) | 289(9) | 3246 |
|  | 18~19 | 360(21.5) | 692(41.3) | 545(32.5) | 310(18.5) | 130(7.8) | 1656 |
| Setting^a^ | Urban | 2819(34.6) | 3633(44.6) | 2347(28.8) | 1583(19.4) | 578(7.1) | 8100 |
|  | Suburban | 2252(27.7) | 3815(47) | 2309(28.4) | 1395(17.2) | 599(7.4) | 8003 |
| School type^a^ | Key school | 1989(31.6) | 3062(48.6) | 1836(29.1) | 1110(17.6) | 296(4.7) | 5761 |
|  | Non-Key school | 3082(31) | 4386(44.1) | 2820(28.3) | 1868(18.8) | 881(8.8) | 10342 |
| Frequency of Self-harm^b, c^ | Never | 3847(75.9) | 6935(93.1) | 4026(86.5) | 2342(78.6) | 713(60.6) | 13918 |
|  | Once | 552(10.9) | 296(4) | 345(7.4) | 296(9.9) | 182(15.5) | 1010 |
|  | Twice or three times | 422(8.3) | 153(2.1) | 204(4.4) | 235(7.9) | 152(12.9) | 643 |
|  | Four times or more | 250(4.9) | 64(0.9) | 81(1.7) | 105(3.5) | 130(11) | 532 |
| Total |  | 5071 | 7448 | 4656 | 2978 | 1177 | 16259 |

^a^ Row percentage.

^b^ Column percentage.

^c^ Gamma test for the association between Frequency of self-harm and age of first drink: *G*=0.338, *P*<0.001 (Boys), *G*=0.507, *P*<0.001 (Girls).

Gamma test for the association between Frequency of self-harm and Drink condition: *G*=0.345, *P*<0.001 (Boys), *G*=0.475, *P*<0.001 (Girls).
