# Supplementary material for: Gender differences in self-harm and drinking behaviors among high school students in Beijing, China
Source: BMC Public Health. 2020 Dec 9;20:1892. doi: 10.1186/s12889-020-09979-6 (PMC7726872; doi:10.1186/s12889-020-09979-6)
Supplement: Supplementary file 2 — Additional file 2: Supplementary Table 2. Results of Log-binomial regression (Reasons for drinking). [file 12889_2020_9979_MOESM2_ESM.docx]

Supplementary Table 2 Results of Log-binomial regression (Reasons for drinking)

| Reasons ^a^ | Total | Girls | Boys | ^b^Interaction term *p* values |
| --- | --- | --- | --- | --- |
|  | AORs | AORs | AORs |  |
| Feeling down | 4.607(4.107, 5.168) | **5.742(5.184, 6.361)** | **4.402(3.916, 4.947)** | **0.008** |
| Customary chronic | 4.063(3.356, 4.919) | **5.460(4.464, 6.677)** | **3.945(3.256, 4.780)** | **0.033** |
| Asked by others | 2.341(1.859, 2.947) | 2.799(2.085, 3.756) | 2.270(1.803, 2.859) | 0.398 |
| No other drinks | 2.204(1.723, 2.817) | 2.849(2.225, 3.648) | 2.163(1.691, 2.766) | 0.161 |
| Feeling delighted | 1.890(1.603, 2.228) | **2.615(2.183, 3.132)** | **1.806(1.531, 2.131)** | **0.016** |
| Drinking with peers | 1.899(1.711, 2.109) | **2.693(2.433, 2.981)** | **1.797(1.616, 1.998)** | **<0.001** |
| Curiosity | 1.782(1.457, 2.179) | 1.733(1.381, 2.174) | 1.794(1.467, 2.194) | 0.861 |
| Others | 1.634(1.320, 2.023) | **2.509(2.110, 2.984)** | **1.588(1.282, 1.967)** | **0.002** |

^a^ Controlling for age, settings and school type, compared with never drink.

^b^ Interaction on gender.
