# Supplementary material for: Gender differences in self-harm and drinking behaviors among high school students in Beijing, China
Source: BMC Public Health. 2020 Dec 9;20:1892. doi: 10.1186/s12889-020-09979-6 (PMC7726872; doi:10.1186/s12889-020-09979-6)
Supplement: Supplementary file 3 — Additional file 3: Supplementary Table 3. AORs for students in different school types by gender. [file 12889_2020_9979_MOESM3_ESM.docx]

Supplementary Table 3 AORs for students in different school types by gender

| Demographic Factors | Total | Girls | Boys | Interaction term^a^ *p* |
| --- | --- | --- | --- | --- |
|  | AORs | AORs | AORs |  |
| Age |  |  |  |  |
| 12~15 | 1.303(1.232, 1.377) | **1.493(1.382, 1.612)** | **1.129(1.040, 1.222)** | **<0.001** |
| Setting |  |  |  |  |
| Suburban | 0.997(0.944, 1.053) | 0.980(0.908, 1.058) | 1.014(0.938, 1.096) | 0.918 |
| School type |  |  |  |  |
| Non-Key school | 1.074(0.990, 1.166) | **1.168(1.078, 1.266)** | **1.058(0.974, 1.149)** | 0.175 |

^a^ Interaction between gender and the demographic factors listed.
